# Bayesian Analysis of Congruence of Core Genes in *Prochlorococcus* and *Synechococcus* and Implications on Horizontal Gene Transfer

**DOI:** 10.1371/journal.pone.0085103

**Published:** 2014-01-21

**Authors:** Nicholas J. Matzke, Patrick M. Shih, Cheryl A. Kerfeld

**Affiliations:** 1 Department of Integrative Biology, University of California, Berkeley, California, United States of America; 2 Department of Plant and Microbial Biology, University of California, Berkeley, California, United States of America; 3 US Department of Energy-Joint Genome Institute, Walnut Creek, California, United States of America; Columbia University, United States of America

## Abstract

It is often suggested that horizontal gene transfer is so ubiquitous in microbes that the concept of a phylogenetic tree representing the pattern of vertical inheritance is oversimplified or even positively misleading. “Universal proteins” have been used to infer the organismal phylogeny, but have been criticized as being only the “tree of one percent.” Currently, few options exist for those wishing to rigorously assess how well a universal protein phylogeny, based on a relative handful of well-conserved genes, represents the phylogenetic histories of hundreds of genes. Here, we address this problem by proposing a visualization method and a statistical test within a Bayesian framework. We use the genomes of marine cyanobacteria, a group thought to exhibit substantial amounts of HGT, as a test case. We take 379 orthologous gene families from 28 cyanobacteria genomes and estimate the Bayesian posterior distributions of trees – a “treecloud” – for each, as well as for a concatenated dataset based on putative “universal proteins.” We then calculate the average distance between trees within and between all treeclouds on various metrics and visualize this high-dimensional space with non-metric multidimensional scaling (NMMDS). We show that the tree space is strongly clustered and that the universal protein treecloud is statistically significantly closer to the center of this tree space than any individual gene treecloud. We apply several commonly-used tests for incongruence/HGT and show that they agree HGT is rare in this dataset, but make different choices about which genes were subject to HGT. Our results show that the question of the representativeness of the “tree of one percent” is a quantitative empirical question, and that the phylogenetic central tendency is a meaningful observation even if many individual genes disagree due to the various sources of incongruence.

## Introduction

The study of bacterial phylogenetics is complicated by the pervasive phenomenon of horizontal gene transfer (HGT), in which gene trees no longer reflect the vertical evolutionary history of cell division due to the incorporation of non-endogenous genes [1]. There is still contention regarding how frequently HGT occurs in nature. In cyanobacteria, per-gene rates of HGT have been estimated to be as low as 16% and as high as 50% [2], [3]. Marine cyanobacteria provide an intriguing case study of HGT, as it is thought that they have undergone a large amount of HGT [4]. The discovery that cyanophages contain laterally-acquired genes associated with the photosynthetic machinery of cyanobacteria provides an attractive mechanism for HGT mediated by phage transduction in marine environments [5].

With such discoveries, the question has arisen: is “tree-thinking” a bankrupt model for understanding evolutionary history, at least in unicellular organisms like cyanobacteria? Doolittle et al. [6] have suggested that tree-thinking may be an inherently flawed way of understanding species relationships due to a bias in assuming relationships in a bifurcating manner rather than in a network [7]. Doolittle has even suggested that “tree thinking is surely a form of typological thinking writ large” [6]. Is it time to abandon the “tree of life” as a metaphor for evolutionary history? Or can the tree of organismal relationships, the tree derived from vertical inheritance of genes through standard cellular replication, still be estimated reliably, despite the horizontal transfer of some genes? However, it is generally accepted that vertical inheritance is vastly more frequent than horizontal transfer, at least if measured on a number-of-gene-replication-events-per-unit-time basis [1]. If this is true, then there is, in a meaningful sense, a tree of organismal relationships which is the product of these vertical replication events, whether or not it can accurately estimated. However, debate remains over significant issues. Alternatives to giving up on the search for the organismal tree exist. One proposal is that so-called “Core Genes”, gene families that are found in the genomes of most or all species, are less likely to undergo HGT. It has been hypothesized and observed that the Core Gene set is less likely to be laterally transferred in gamma-proteobacteria [8]; thus, if Core Genes exist in the clade of interest, and can be reliably identified, they would provide a reasonable basis for estimating bacterial species trees.. However, many have expressed their concerns about capturing phylogenetic relations based on concatenating a few well-conserved genes, a “Tree of one percent” [9], and that the tree of one percent many not be meaningful, if the history of most of the genome is dominated by HGT.

One option to assess the representativeness of a tree inferred from concatenation of a few well-conserved genes would be to attempt to detect HGT events in the rest of the genome. There are many different ways through which HGT can be identified, such as GC content, syntenic regions (genomic islands), phylogenetic incongruence, and many others [10]. However, except for phylogenetic incongruence, the other hallmarks of HGT will usually be difficult to detect if the event is ancient, and thus most work on the estimation of the prevalence of HGT focuses on phylogenetic incongruence as the detection method.

Phylogenetic incongruence is defined most simply as disagreement between trees – traditionally, the term is used to refer to disagreements in tree topology, but can also refer to disagreements in branch length. However, incongruence can be measured and represented in many different ways. Likelihood-based tests, such as the Shimodaira-Hasegawa (SH) test [11], [12] and Approximate Unbiased (AU) test [13], are in common use in the literature, and provide a means to test the support of data for different tree hypotheses and infer incongruence between trees. Another method that has been employed in multiple studies is the quantification of conflict between genes by calculating congruent and conflicting topologies among quartets of taxa, and displaying the results with heat-maps [3], [14].

A serious difficulty for any incongruence-based method is that there may be many sources of statistically significant phylogenetic incongruence, even in the hypothetical situation where absolutely no HGT has taken place. These include misestimation of the phylogenetic relationships of genes due to model-misspecification or misestimation, saturation of sequence divergence leading to insufficient phylogenetic signal, mistakes in sequence alignment, long-branch attraction, confusion of synapomorphies and plesiomorphies through mistaken rooting of the tree, and estimation errors due to missing taxa. Apart from incongruence due to these sorts of avoidable and unavoidable errors, “true” incongruence in gene trees can result from common non-HGT processes, such as incomplete lineage sorting, concerted evolution, homoplasy resulting from parallel evolution and convergence (at the molecular level, this may occur e.g. with adaptation to temperature), and, especially, mistaken identification of paralogous genes as orthologous. Thus, even if statistically significant amounts of phylogenetic incongruence are observed, this does not prove that HGT is rampant; it is possible some other non-HGT factor explains some or all of the incongruence. In that hypothetical situation, non-HGT sources of noise are to blame for observed incongruence, and it might be defensible to attempt to compensate for this noise by, for example, concatenating a large number of genes so as to average out the noise.

There are additional reasons why high rates of detectable phylogenetic incongruence might not add up to the impossibility of estimating an organismal or species tree. If horizontal transfer is sufficiently rare, many gene trees may accurately record significant parts of the organismal, vertical tree, even if most genes have experienced horizontal transfer events at some point(s) in their history, and thus an organismal tree might be realistically estimable, even if none of the genes followed it perfectly. Furthermore, even if horizontal gene transfer is not rare at all, if there is organismal phylogenetic signal in the horizontal transfer events themselves – such that HGT events occur much more commonly between close organismal relatives than distant ones – then gene trees may tend to retain substantial organismal phylogenetic signal, despite ubiquitous HGT. (In fact, such situations are extremely common – they are more common known as classic sexually reproducing multicellular species, where ubiquitous genome recombination in every generation nevertheless produces a very treelike phylogenetic pattern because of the limitation of recombination to close relatives.)

Given these complexities, it would be helpful for researchers studying HGT to have a method that assesses the degree of incongruence between the trees of gene families, and thus distinguish between small and large incongruences. It would also be helpful to be able to visualize the relative similarities and differences of a large number of gene trees, to see what if any common phylogenetic patterns emerge, and to what extent incongruence blurs it. As a step towards this end, we address the following issue: given a large core gene dataset, consisting of a large number of gene families shared across some taxa of interest, how should researchers determine whether or not it is reasonable to attempt to estimate the tree of organismal relationships through concatenation of a few well-conserved genes?

We focus our efforts on improving our understanding of the degree of vertical phylogenetic signal in the Core Gene dataset in the marine cyanobacterial subgroups *Prochlorococcus* and *Synechococcus*. Marine cyanobacteria provide an ideal test case for HGT, because some studies have detected HGT between taxa correlating with phylogenetic relatedness and with the non-phylogenetic geographical/physical proximity [15]. Moreover, they are a globally relevant group of bacteria that provide significantly to primary production in oligotrophic regions of the ocean [16]. As all cyanobacteria are able to perform oxygenic photosynthesis, many Core Genes are part of large protein complexes that are involved in this process such as Photosystem II, the Cytochrome b6f complex, Photosystem I, ATP Synthase, and the carboxysome. Furthermore, many cyanobacterial species have been sequenced, and in particular the marine genera *Prochlorococcus* and *Synechococcus* have been heavily sampled. This provides abundant genomic information and improves the chance of distinguishing HGT from other causes of phylogenetic incongruence.

We identify a set of 379 “Core Genes” from *Prochlorococcus* and *Synechococcus*. For each gene family, the sequences are aligned and the posterior distribution of trees is estimated, producing a “treecloud” [17], [18] for each alignment. The same was done with a putative organismal tree that was generated from a concatenated alignment of a smaller subset of the Core Genes made up of 31 informational proteins widely conserved across the 3 domains and used by [19]. We will refer to these as “Universal Proteins” (hereon referred to as UP). We then use tree-to-tree distance metrics to estimate the degree of incongruence between the tree distributions derived from each of the 380 alignments. We use non-metric multidimensional scaling (NMMDS) to visualize the resulting 380×380 all-versus-all matrix of average tree-to-tree distances within and between treeclouds [20]. This allows rapid assessment of the overall level of incongruence between individual gene families and between the gene families and the organismal tree (i.e. the UP tree), and to identify significant and nonrandom patterns in the degree of incongruence exhibited by each gene family.

In addition to the visual heuristic of NMMDS plots, we analyze the treecloud-to-treecloud distances matrices and demonstrate that the UP tree is indeed at or very near the statistical center of the treespace occupied by the 380 treeclouds and furthermore that the 380 treeclouds cluster in a tiny region of treespace compared to that occupied by a population of 380 treeclouds where phylogenetic signal has been degraded due to randomization of the tip labels.

Our results show that, despite the fact that moderate amounts of incongruence between treeclouds (and within treeclouds) is ubiquitous, it is nevertheless true that Core Gene trees rarely strongly conflict with the organismal tree.We also compare the SH- and AU-tests for incongruence with the tree-to-tree distance metrics, and Ge et al.'s (2005) gamma statistic, and show that these methods identify few cases of HGT; furthermore, they disagree about which genes were subject to it. We suggest that studies of HGT should be careful to consider non-HGT sources of phylogenetic incongruence, and that calculations of treecloud distance matrices, combined with NMMDS methods and statistical characterization of the clustering in treecloud space and the “centrality” of a Universal Protein tree, may prove valuable as a means of assessing whether a large multigene dataset has a central tendency towards a tree of organismal relationships – similar to Ge et al.'s (2005) “cobweb of life” concept – or is so ridden with various sources of incongruence that the organismal tree cannot be safely estimated.

## Materials and Methods

### Identification of Core Gene Families and Generation of Core Gene Trees

Total protein coding sequences for each genome were retrieved using IMG [21]. 28 species were used for analysis, including *Prochlorococcus marinus* strain AS9601, *Prochlorococcus marinus* strain MIT 9211, *Prochlorococcus marinus* strain MIT 9215, *Prochlorococcus marinus* strain MIT 9301, *Prochlorococcus marinus* strain MIT 9303, *Prochlorococcus marinus* strain MIT 9312, *Prochlorococcus marinus* strain MIT 9313, *Prochlorococcus marinus* strain MIT 9515, *Prochlorococcus marinus* strain NATL1A, *Prochlorococcus marinus* strain NATL2A, *Prochlorococcus marinus* marinus strain CCMP1375, *Prochlorococcus marinus* pastoris strain CCMP1986, *Synechococcus* sp. BL107, *Synechococcus* sp. CC9311, *Synechococcus* sp. CC9605, *Synechococcus* sp. CC9902, *Synechococcus* sp. RCC307, *Synechococcus* sp. RS9916, *Synechococcus* sp. RS9917, *Synechococcus* sp. WH 5701, *Synechococcus* sp. WH 7803, *Synechococcus* sp. WH 7805, *Synechococcus* sp. WH 8102, *Synechococcus elongatus* PCC 6301, *Synechococcus elongatus* PCC 7942, *Synechococcus* sp. JA-2-3Ba(2-13), *Synechococcus* sp. JA-3-3Ab, *Synechococcus* sp. PCC 7002. Reciprocal BLAST methods, with a threshold set at 10^−6^, were used to determine orthologous genes. As a generic method for identifying orthologs, reciprocal BLAST is dubious, but here we kept only gene families that contained one significant hit per genome (single-copy orthologs), thus dramatically reducing the risk of mistakenly identifying orthologs as paralogs. (Although this procedure does not completely eliminate the possibility of such mistakes, for example if an ancestral gene duplication takes place and only one or the other of the duplicate pair of genes survives in each sampled species, this should be relatively rare as it requires multiple coincidental events. In any case, such events are just the sort that can produce gene trees in conflict with the organismal tree, and thus be mistakenly identified as HGT events.) Thus, gene families that contained an ortholog in all genomes and lacked the presence of any paralogs (defined by more than one BLAST hit in a genome) were defined as Core Gene families. Eliminating gene families with known paralogs decreases the chances of hidden paralogy [22]. With these criteria, 379 Core Gene families were identified.

Fasta format sequences for all Core Gene families were collected, and each family was aligned using MAFFT, using the –maxiterative function [23]. Gene trees were estimated using MrBayes 3.1.2 [24], with the amino acid model set to sample from the 10 available substitution models (with equal prior probability on each model), with the proportion of invariant sites and the base frequencies estimated. Each analysis used two independent runs (4 chains each) and was run for 1 million generations, with every 1000^th^ tree sampled. The first 50% of each run was discarded as burn-in, leaving a total of 1000 trees as the sample from the posterior distribution of trees for each gene family. The majority-rule consensus tree was calculated for each posterior distribution as the best point estimate of the tree, although the posterior sample of trees was used for all distance calculations. All 379 MrBayes analyses were checked for convergence; using the standard convergence diagnostic of standard deviation of split frequencies between the two independent runs, 99% (375/379) of the runs convergence to values of less than 0.1 after 1,000,000 generations, and 93% less than 0.02. The maximum value of the convergence diagnostic was 0.125 (1/379 trees). If some gene families had difficulty reaching a very low convergence diagnostic due to conflicting signal or lack of signal, this would itself be an indication of a possible source of incongruence in that gene family, resulting in a more spread out distribution of posterior trees. Our research objective mandated that we take this kind of uncertainty into account. Each posterior distribution of trees was summarized with a combinable consensus tree displaying posterior clade credibility for each resolved branch (however, the posterior distribution of trees from each analysis, not the consensus tree, was later used to calculate average tree-to-tree distances). Core Gene protein sequences were retrieved by using their IMG object identifier and were assigned to functional categories based on Clusters of Orthologous Groups (COGs) using IMG.

### Generation of the Universal Ribosomal Protein Tree

**Figure 1:**
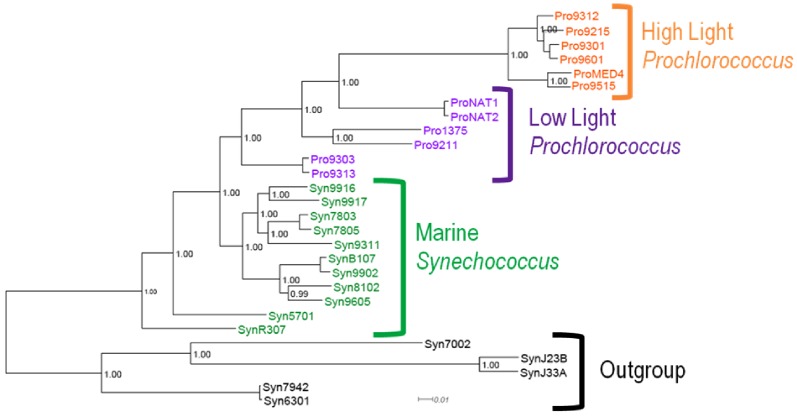
Phylogeny of the cyanobacterial species included in this study, with major subgroups highlighted. This well-resolved phylogeny is a majority-rule consensus tree from MrBayes analysis of the concatenated alignment of the 31 proteins in the UP dataset. Posterior probabilities of each bipartition in the tree are shown at the top of each branch.

A species treecloud was generated by concatenation of thirty-one conserved ribosomal proteins(*dnaG*, *frr*, *infC*, *nusA*, *pgk*, *pyrG*, *rplA*, *rplB*, *rplC*, *rplD*, *rplE*, *rplF*, *rplK*, *rplL*, *rplM*, *rplN*, *rplP*, *rplS*, *rplT*, *rpmA*, *rpoB*, *rpsB*, *rpsC*, *rpsE*, *rpsI*, *rpsJ*, *rpsK*, *rpsM*, *rpsS*, *smpB*, and *tsf*), as previously described by Wu et al. [19]. Homologs of each ribosomal protein were identified using reciprocal BLAST of the 49 publicly available cyanobacterial genomes in IMG at the end of 2009. These gene families were aligned as described above and the subsequent alignment was used to create Hidden Markov Models (HMMs) for the respective ribosomal protein using HMMer v.2.0 [25]. Using HMMer, the hmmsearch function was used to identify orthologs and align them using the hmmalign function. The resulting thirty-one alignments were then concatenated. The final concatenated alignment was used to generate a distribution of trees using MrBayes, using the same settings as used for the individual gene trees from the Core Genes dataset (described above). The majority-rule consensus tree of this treecloud is shown in Figure 1.

### Testing for incongruence

Three statistical tests were used to assess the degree of phylogenetic incongruence between treeclouds estimated from the sequence data: the SH-test [11], [12], the AU-test [13], and Ge et al.'s [10] gamma. The SH-test and AU-test compare two or more topologies, at least one of which was derived from optimization on the sequence data in question. The null hypothesis for these tests is that all topologies are equally good explanations of the data. To reject the null hypothesis, the difference in likelihood between the topology with maximum likelihood and an alternate tree must be statistically significantly higher than the difference between the highest likelihood and the lower likelihood calculated on the two topologies for a set of bootstrap sequence datasets generated through resampling. The SH-test has been found to be conservative, so as an improvement Shimodaira devised the AU-test, which uses multiscale bootstrapping to assess how the p-value for significant difference in likelihoods changes with different amounts of bootstrap-produced sequence, and corrects accordingly. The SH- and AU-tests were calculated using CONSEL [26] on the site-likelihoods calculated with Tree Puzzle 5.2 [27], -wsl option (method described at: http://www.is.titech.ac.jp/~shimo/prog/consel/quick.html). Here, the model of sequence evolution was chosen to be as close as possible to the model with the highest posterior credibility in MrBayes analyses (i.e., WAG [28], which was preferred in over 90% of posterior samples across all 380 MrBayes analyses, with the chloroplast cpREV model [29] taking up most of the rest of the posterior distribution on models).

For both tests, the fully-resolved UP consensus tree was compared to (1) the consensus tree for each gene family, which was often but not always fully resolved and (2) the last tree sampled from the posterior tree distribution for each gene family. Both trees were included as a precaution: trees sampled from the posterior are always fully-resolved, which provides a potentially better alternative tree in the event that a gene family's incompletely-resolved consensus tree contains artifacts that move it far away from the (formally unknown) true optimal topology.

Ge et al.'s [10] gamma was calculated using the *treedists* function in PAUP* [30], automated with an R script. Their gamma statistic takes branch-transfers into account as well as generic incongrunce as measured by tree-to-tree distances. Ge's gamma statistic is given below, where *T* and *T′* represent the two trees, *m* and *n* are the number of branches in the two trees, *x* is the number of taxa, and *d_S_* and *d_M_* are the symmetrical distance and MAST distance (i.e. SPR distance, number of subtree pruning and regrafting steps; [30]), as calculated in PAUP*:
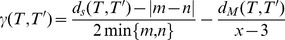
(1)The equation thus consists of the normalized symmetric distance between two trees minus the normalized SPR distance. The former is a general measure of overall incongruity; the latter measures how many SPR events would be required to bring the topologies of the two trees into congruence. Ge et al. proposed that a pair of trees with a large symmetric distance, but small SPR distance, which would therefore have a high gamma statistic, would represent the strongest case for an HGT event, in that not only was there a large amount of incongruence between two trees, but it could be removed with only one or a few branch-swapping events (which approximates what happens to phylogenetic structure when a gene is replaced by a laterally-transferred ortholog). The null distribution on gamma was calculated on 1000 random pairs of trees randomly sampled from the last 50% of the posterior sample of trees estimated for each gene family. Then, gamma was calculated on 1000 tree pairs randomly chosen between the gene family treecloud in question and the UP treecloud (only trees from the last 50% of the posterior sample were sampled for both treeclouds). Each of the between-treecloud gammas was ranked against the 1000 within-treecloud gammas. The number of those 1000 between-treecloud gammas that fell at or above the 95^th^ percentile of the null gamma distribution was counted. This count was then compared against the null expectation using a binomial null distribution where only 5% of the 1000 between-treecloud gammas would be expected to lay in the top 5% to produce a p-value.

### Calculation of tree-to-tree distances

For each of the 380 posterior distributions of trees (“tree clouds”) derived from MrBayes, the average pairwise distance to every other tree cloud was calculated. Two tree-to-tree distance metrics were calculated using DendroPy [31]: 1) the Robinson-Foulds branch-length distance, the summation of the squares of the differences in branch lengths between two trees, and 2) the symmetric distance, the tree distance in topology space, ignoring branch lengths, in which a branch present in one tree and absent in the other results in a distance of 1. As each post-burnin tree cloud contains 1000 trees, calculating the tree-to-tree distances for each possible pair was computationally infeasible; thus 100 trees were randomly selected from each tree cloud, and the distance calculated for each pair.

The total treelength (expected number of substitutions per site; high substitution rate = high total treelength) is expected to heavily influence the Robinson-Foulds branch-length distance: e.g., two small trees will have a much lower RF distance between them than the same two trees with branch lengths multiplied by 10. Thus, the original collection of 380 posterior distributions of trees was copied, and every sampled tree was normalized to total treelength = 1. Then RF branch-length distance was calculated again for this set of distances.

This distribution of distances was summarized with a mean and standard deviation. As a measure of the spread of the trees within each posterior distribution, the average tree-to-tree distance *within* each tree cloud was also calculated, using the same methods as above. The tree-to-tree distance was also summarized as the ratio of the mean between-treecloud distance divided by the mean within-treecloud distance, as a normalized measure of the difference between trees from different treeclouds and the same treecloud.

### Histograms and T-tests

To develop a test for the significance of differences between posterior distributions of gene trees and the posterior tree distribution of the UP dataset, we compared two distances: 1) the within-treecloud distances for each gene tree and 2) the gene-tree-to-UP-tree distances (from here on referred to as the between-treecloud distance). The significance of the difference in means was assessed with a one-tailed Welch's t-test (which allows for unequal variances in the samples) with Bonferroni correction for multiple tests. The between- and within-tree distances were also checked for significant overlap by calculating whether or not the 92^nd^ percentile of the within-treecloud distances was higher than the 8^th^ percentile of the between-treecloud distances. If so, the distributions were scored as “non-overlapping”. The percentiles used are based on the 84% confidence intervals of the distributions, which are the confidence intervals recommended by Payton et al. [32] to appropriately control for error when using confidence intervals to assessing the overlap of two distributions with approximately equal-sized confidence intervals.

Results from the t-test of within- and between-treecloud distances, and the raw between-treecloud distances, were compared to the SH-test, AU-test, and Ge's gamma results to see if the same or similar gene families were identified as being incongruent with the UP treecloud.

### Non-metric multidimensional scaling (NMMDS)

To visualize the very large space of a 380×380 distance matrix in 2 dimensions, treecloud-to-treecloud average distances were transformed with NMMDS as implemented in the *nmds* function of the R package vegan [33]. Application of this method to a space of phylogenetic trees was pioneered by Hillis et al. [20]. Briefly, NMMDS represents the high-dimensional space in 2 dimensions for visualization, attempting to minimize a stress function [34], [35]. This technique displays more information about the multidimensional space, and potential clustering in that space than, for example, representing a multidimensional dataset in 2-dimensions by simply plotting the first two components of a Principal Components analysis. However, any 2D representation of a hyperdimensional space will be a simplified abstraction of the original space, and different random seed values will produce somewhat different representations, so every NMMDS was produced 3 times from different seeds so as to represent this variability for the viewer.

NMMDS was run on the following mean between-treecloud distances: RF branchlength distances calculated on the raw treeclouds (not rescaled to account for variation in total treelength); symmetric distances; and RF branchlength distances calculated on the treeclouds after each tree had been rescaled to a total treelength of 1.

The distribution of variables that might correlate with congruence/incongruence for non-HGT reasons was explored. The variables used were treelength, alignment length, and (alignment length/treelength). The variables were displayed on the NMMDS plots by represented via color gradient, with red representing low values and yellow representing high values. The UP treecloud was represented with a star. The correlation of these variables with treecloud-to-treecloud distances and various measures of incongruence was explored via linear regression.

### Simulation of treeclouds with no phylogenetic signal

NMMDS visualizations have the limitation of no absolute scale. It would be useful to give the user a sense of what the treecloud-to-treecloud space would look like if gene treeclouds were included with similar statistical properties to the observed treeclouds, but with no congruence with the observed trees other than produced by chance. To that end, treeclouds with completely randomized phylogenetic signal [36], [37] were generated by taking each tree from the original analysis and randomly reshuffling tip labels, then adding these trees to the original dataset. Distances and NMMDS plots were calculated, as above, for this enlarged dataset of 760 treeclouds.

### Statistical test of the centrality of the UP treecloud in the hyperdimensional treecloud space

For each gene treecloud, the average distance to every other treecloud was calculated by randomly sampling 1000 pairs of trees and calculating the tree-to-tree distance for each. This resulted in 379 average distances. The distribution of these average distances was Gaussian and was taken as the null hypothesis. If the UP treecloud does not represent the central phylogenetic tendency of the gene treeclouds, then the UP treecloud might be expected to have the same distribution of distances to other treeclouds as the gene treeclouds, and the same mean distance to other treeclouds. The average distance between the UP treecloud and the gene treeclouds was calculated by taking the mean of 1000 randomly-sampled pairs of trees. The rank of the mean between-UP-and-gene treecloud distance on the null, divided by 379, was taken as the p-value. This calculation was performed on the treeclouds of symmetric distances and of RF branchlength distances on rescaled trees. To check whether or not the inclusion of individual UP treeclouds in the 379 gene treeclouds influenced the statistic, the individual UP treeclouds were removed and the calculation was repeated on the 354 remaining treeclouds.

### Summarizing similarity of gene treeclouds to the UP treecloud

The similarity of a collection of gene treeclouds to a reference treecloud – in this case the UP treecloud – can be thought of as the “treeishness” of the dataset, and summarized with a 1-dimensional treeishness statistic, τ. τ is derived by calculating the relative distance of the population of gene treeclouds to the central UP treecloud, versus the random treeclouds:
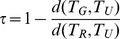
(2)where *d*(*T_G_*,*T_U_*) represents the distance between a sample from the posterior distribution of a gene treecloud and a sample from the posterior distribution of the UP treecloud, and *d*(*T_R_*,*T_U_*) represents distance between a sample from a tips-randomized gene treecloud and a sample from the posterior distribution of the UP treecloud. If the gene treecloud was no more similar to the UP treeclound than the randomized trees, τ would equal 0; if the gene treeclouds were identical to the UP treecloud, with tree distances of 0, then τ would equal 1. The treeishness can be calculated for an individual triplet of trees (a gene tree, a UP tree, and a tips-randomized tree), or for a large sample of such triplets, or using the means of the distances between these categories of treeclouds. Both the sampling and means strategies were used, producing essentially identical values of τ. The sampling strategy also produced a distribution of τ values, which was used for testing whether or not τ was significantly different from 0 and 1. The τ statistic was calculated using both symmetrical (topology) distances and RF-branchlength distances using the rescaled trees, and both with and without the individual UP gene treeclouds.

## Results

### Phylogenetics of the UP dataset

The consensus phylogenetic tree based on the UP dataset is shown in Figure 1. The tree exhibits very high resolution, as is common for large concatenated datasets. Our UP tree is consistent with previous work [38], finding phylogenetic separation with high support between high-light and low-light *Prochlorococcus*.

### Visualization of treecloud-to-treecloud distances using NMMDS methods

Manually assessing the similarities and differences between hundreds of gene trees and the UP tree would be an onerous and subjective task. (Example trees based on alignments of single genes are shown in Figure S1 in File S1.) In addition, while branch-support values, such as the posterior clade credibility values and bootstrap support values, are commonly displayed on consensus trees, and give the user a sense of the support that the data lend to that portion of the tree, there is not a standard method for interpreting branch support values for or against a particular hypothesis of HGT.

**Figure 2:**
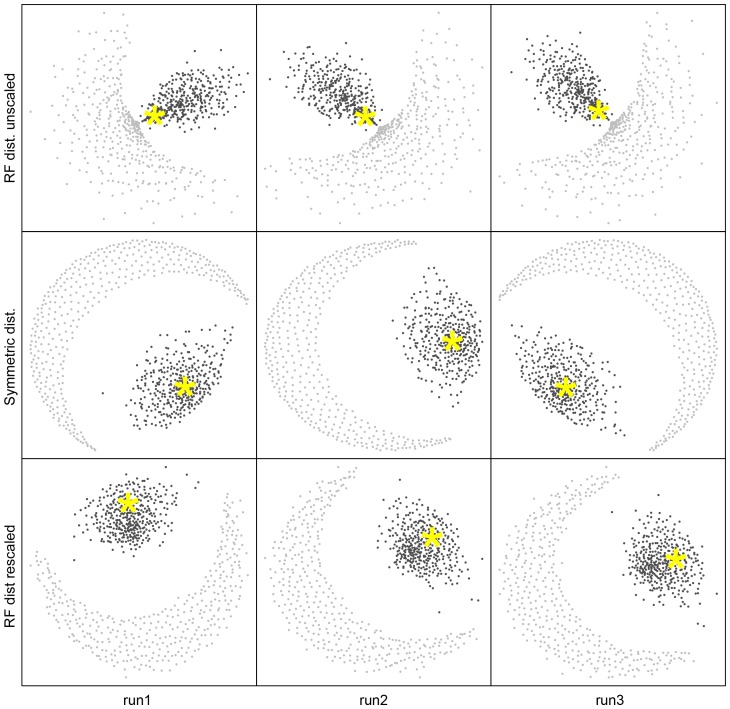
Non-metric multidimensional scaling (NMMDS) plots, representing in two dimensions the distances between 760 treeclouds. Black dots represent the 380 treeclouds resulting from MrBayes analyses of 379 single-protein alignments and the UP alignment. Grey dots are 380 randomized treeclouds produced by randomly reshuffling the tip labels of each MrBayes tree. Top row: NMMDS plots of the mean Robinson-Foulds (RF) branch-length tree distances between treeclouds. Middle: NMMDS plots of the mean symmetric topology (sym) differences between treeclouds. Bottom: NMMDS plots of the mean RF distance between treeclouds when each tree was rescaled to a total length of 1. The three columns are equivalent and represent three different NMMDS runs starting from three different random seeds. The star represents the treecloud of the UP dataset.

However, a rapid assessment of the overall congruence of the gene trees with the UP tree can be made with NMMDS plots (Figure 2). It is immediately evident from inspection that the central pattern in the NMMDS plot is that a large number of the individual gene family treeclouds cluster near each other and near the UP treecloud. Indeed, this appears to be the only major structure that NMMDS recovers in the treecloud distance matrix. The results of three independent NMMDS runs are shown in Figure 2 as a reminder that orientation, direction, etc. of NMMDS plots is arbitrary. The examination of several independent NMMDS runs is recommended to be sure that visually apparent clustering patterns are consistently observed.

### Inclusion of treeclouds lacking phylogenetic signal in NMMDS plots

A possible concern with using the NMMDS plot to reach the conclusion that there is a central tendency towards a common phylogenetic pattern could be stated as follows. NMMDS plots are scale-free, only giving information about the relative distances between treeclouds, and only giving approximate information on even the relative distances. There is no way to tell whether or not most of the treeclouds share a common phylogenetic signal, or whether just the treeclouds right in the central cluster share this signal, with the rest of the treeclouds having only random relationship to the central cluster.

This concern is addressed by taking all 380 treeclouds and randomizing the leaves of each tree and including them in the plot (in grey) as shown in Figure 2. As expected, the completely-randomized treeclouds all plotted at the very periphery of the NMMDS plot, giving some sense of what would be expected if a large number of genes had no congruent signal for some reason (e.g., lack of sequence similarity due to misalignment or misattribution of homology; or, perhaps, massive amounts of HGT, at least if the HGT between taxa happened with absolutely no correlation with phylogenetic relationship). Moreover, various degrees of complete randomization also displayed this similar trend of pushing out towards the periphery (Figure S2 in File S1). It appears that distance-based methods are a clear and direct technique useful for assessing the central phylogenetic tendency of a large number of gene histories.

### Location of individual UP treeclouds in the NMMDS plots and validation of the NMMDS plots

**Figure 3:**
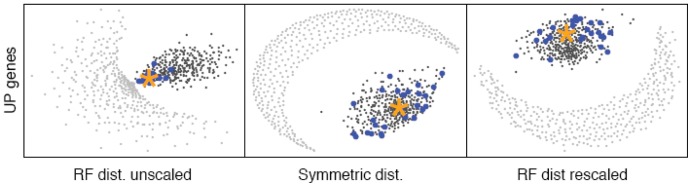
Distribution of individual UP gene tree treeclouds in treespace (blue dots). Other symbols as in Figure 2.

Another possible concern with the NMMDS plot is that the observed clustering with the UP tree might be due entirely to the clustering of the individual universal proteins with the concatenated UP tree, since universal proteins were included in the 379 single-protein gene families as well as the UP concatenated dataset. However, this concern can be assessed by plotting the location of the individual UP treeclouds on the NMMDS plot (Figure 3). While it is clear that the treeclouds of protein alignments from the universal protein list exhibit significant clustering with the concatenated UP treecloud, it is also clear that many non-UP proteins exhibit this same clustering.

The importance of taking into account the variation in overall treelength is highlighted in the top row of Figure 2, which shows the NMMDS plot of RF branchlength distance calculated on raw trees with no rescaling of total treelength. Because the RF metric takes into account branch length, when comparing trees with very short lengths (eg. slowly evolving genes), the trees will have a very small tree-to-tree RF distances, even though they may not reflect similar topologies. This is shown in the top row of Figure 2 (and Figure 3, left), where some of the tip-randomized treeclouds cluster very close to some of the gene treeclouds and the UP treecloud. It is obvious that interpretation of tree-to-tree RF branchlength distance results will be overwhelmingly influenced by variation in total treelength, and we warn against naïve application of RF branchlength distances without taking this factor into account. The treelength variation was successfully corrected when all gene trees were rescaled to a total treelength of 1, as shown in the NMMDS plots of RF branchlength distances between treeclouds with rescaled trees (Figure 2, bottom row; Figure 3, right).

When interpreting NMMDS plots, it is important to remember that a high-dimensional space has been flattened into a 2-dimensional space for display, and thus the plots only provide an approximation of the true treespace. For example, a naïve interpretation of the NMMDS plots of symmetric distances between treeclouds and RF-distances between rescaled treeclouds in Figures 2 and 3 might suggest that the tips-randomized treeclouds (grey dots) are clustering with each other. In reality, they are all approximately equidistant from each other and from the non-randomized treeclouds. (The average distance between tips-randomized treeclouds is approximately 50 for symmetric distance, and about 2.0 for the RF branchlength distance when the trees have all been rescaled to treelength = 1.) The NMMDS algorithm detects the clustering of the core gene treeclouds with each other, and the equidistance of the randomized trees from each other and from the core gene treeclouds. An exact representation is impossible in 2-dimensional space, so the randomized treeclouds are instead displayed in an arc where all the randomized treeclouds are displayed approximately the same distance from the core gene cluster.

The NMMDS algorithm returns a stress value, which gives a relative measure of the distortion required to transform multidimensional distance space into a 2-dimensional plot, and an r^2^ value which measures the amount of actual variation in the high-dimensional space which is accounted for by the variation in the 2-dimensional plot. Table 1 shows these summary statistics. The three iterations were all very similar. Inclusion of the reshuffled tips-randomized trees in the plots tended to increase the stress, but only slightly, and correspondingly tended to decrease r^2^ by about 0.05–0.08. While inclusion of the randomized treeclouds makes for a somewhat more difficult NMMDS optimization problem, major distortions of the depiction of the gene treecloud space do not appear to be a result. The RF-distances on the unscaled treeclouds have much higher r^2^ than the other analyses; this is due to variation in total treelength being a variable in common across all 380 unscaled treeclouds which strongly correlates with RF-distance.

**Table 1:** Summary statistics for NMMDS analyses.

		Stress value			r^2^		
Distance measure	Tips-randomized trees included?	Iteration #1	Iteration #2	Iteration #3	Iteration #1	Iteration #2	Iteration #3
RF-dist., unscaled	Yes	0.27	0.27	0.27	0.75	0.75	0.75
RF-dist., unscaled	No	0.24	0.24	0.24	0.83	0.83	0.83
Symmetric distance	Yes	0.36	0.36	0.36	0.50	0.50	0.50
Symmetric distance	No	0.35	0.34	0.34	0.55	0.56	0.56
RF-dist., rescaled	Yes	0.35	0.35	0.35	0.54	0.54	0.54
RF-dist., rescaled	No	0.34	0.34	0.34	0.60	0.60	0.59

### Correlates of incongruence: Complexity hypothesis, effect of alignment length and divergence on tree distance

We found no support in this dataset for the Complexity Hypothesis, i.e. the idea that genes with core informational functions will be less subject to HGT than more optional metabolic genes (Figure S3 in File S1). This may be due to the fact that evidence for HGT in this dataset appears to be weak overall; these results are discussed further in Text S1. We did find numerous correlates of incongruence with alignment length and tree length; these confirm what would be predicted from first principles: shorter alignments and longer trees both exhibit more incongruence (Figures S4, S5, S6 in File S1). Results and plots illustrating these effects are shown in Text S1.

### Comparison of different methods in detecting HGT

In addition to symmetric and branch-length tree distances, we used three additional methods for detecting phylogenetic incongruence and possible HGT: Two likelihood-based methods (the SH-test and the AU-test) and a branch-transfer-based method, Ge et al.'s (2005) gamma statistic. All three methods, plus our distance-based approach were implemented on our 379 core gene trees and UP tree in order to identify candidate gene trees that do not reflect the same evolutionary history as the UP tree.

**Figure 4:**
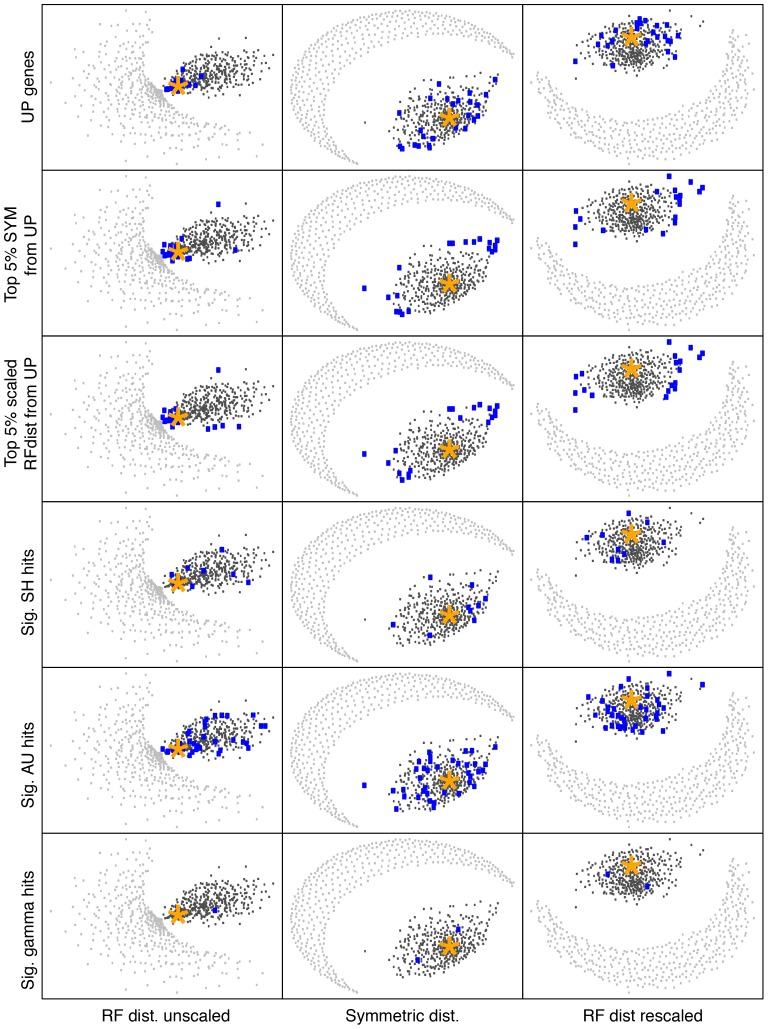
Five metrics of incongruence visualized with NMMDS plots. Treeclouds identified as highly incongruent are shown by blue dots.

As both likelihood-based tests are used frequently to identify phylogenetic incongruence that may indicate HGT, it was natural then to compare our distance-based measures of incongruence. However, this yielded surprising results. For example, when ranking treeclouds based on their degree of incongruence with the UP tree, as measured by the SH-test or by RF branchlength distance, we find no overlap between trees significantly different from the UP tree picked up by the SH test, and the top 5 percent most-incongruent chosen by RF distance. When the top candidates for significant incongruence are displayed on the NMMDS plot, the differences are evident (Figure 4).

The most significant cases of gene tree/UP tree incongruence as identified by each statistic are labeled on the NMMDS plots in blue. However, it appears that the SH-test, though commonly used as a test of tree congruence, is identifying something much different than incongruence as measured by tree-to-tree distances. The same trend is observed when comparing HGT candidates from the AU test. When implementing Bonferroni correction to the AU-dataset, 37 trees significantly differed from the UP tree, only three trees also picked up from our distance method. Not surprisingly, eight of the nine SH-test candidates were also picked up from the AU-test results, thus, confirming how both likelihood methods are similar but the SH-test appears to be more stringent.

We compared the above methods with Ge et al.'s (2005) gamma statistic for use in identifying possible HGT events. Ge et al. validated their statistic with simulations, and used it to identify incongruent genes that that were likely to be explainable through one or a few branch-transfer events. Using their method, we find only two trees that are identified as potential HGT under Ge et al.'s gamma statistic. The ambiguous results of the other statistics and the low number of candidates identified by Ge et al.'s gamma seem to support the idea that Core Genes undergo relatively low amounts of HGT within the *Prochlorococcus* and *Synechococcus* groups.

The difference in mean distance within gene treeclouds and between gene treeclouds and the UP treecloud were virtually always significantly higher between gene treecloud and UP treecloud, than within a gene treecloud. This is not unexpected, as the posterior distribution of trees is optimized on the alignment for each protein family. For symmetric distance, 374/379 gene treeclouds exhibited a statistically significant difference in within-treecloud and between-treecloud means, even after Bonferroni correction. For RF branchlength distance on rescaled trees, the number was 373/379. However, although the means of within- and between-treecloud distances were almost always significantly different, the within-treecloud and between-gene/UP treecloud distance distributions were nevertheless overlapping more often than not. For the symmetric distances, the within- and between-treecloud distance distributions overlapped in 266/379 gene treeclouds. For the RF branchlength distances, overlap occurred for 316/379 treeclouds.

**Figure 5:**
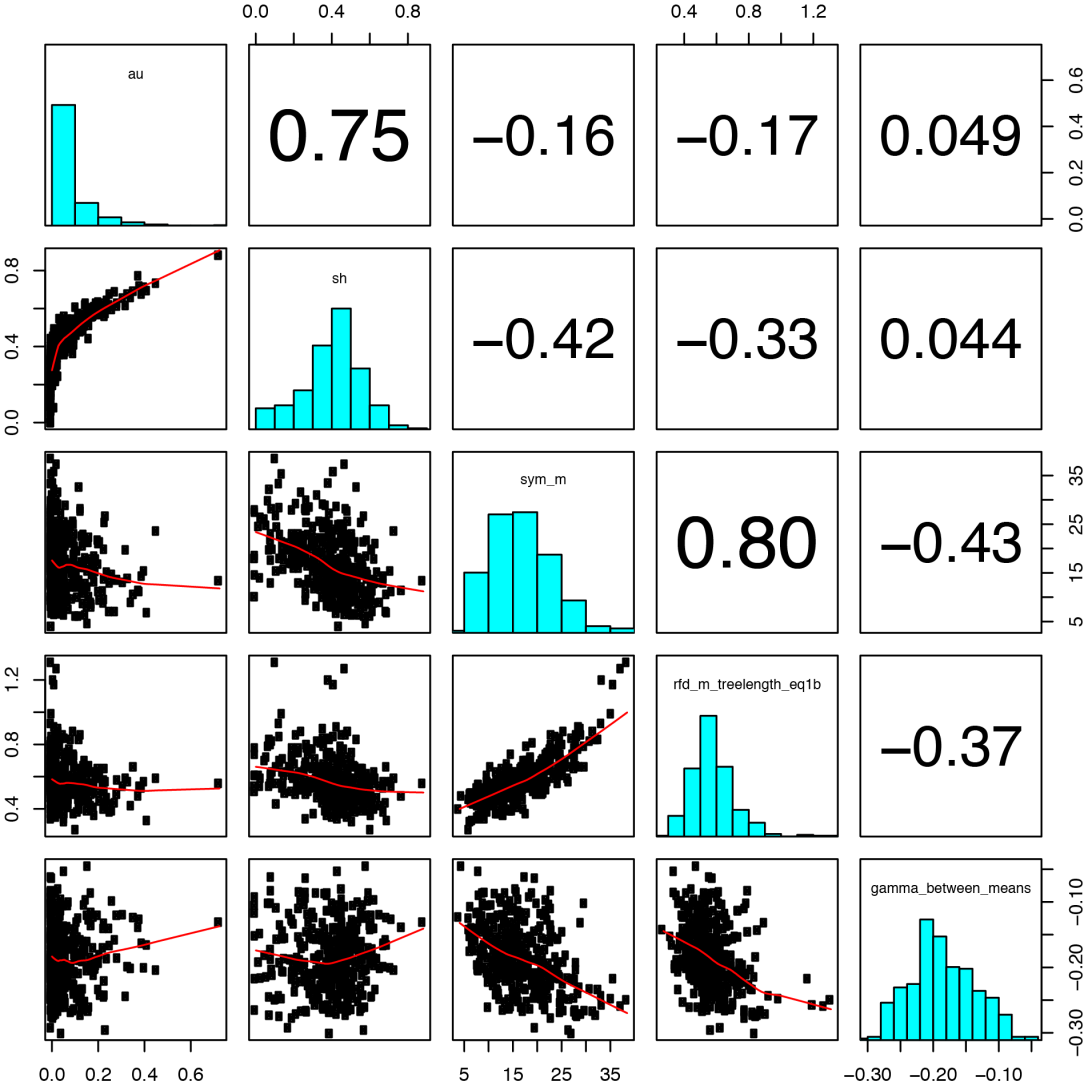
Pairwise comparison of the five metrics of incongruence. In the scatterplots, each variable makes up the x-axis of its column, and the y-axis of its row. The variables plotted, by column, are (1) AU-test p-values; (2) SH-test p-values; (3) Symmetric distance to the UP treecloud; (4) RF-branchlength distance (rescaled) to the UP treecloud; and (5) Ge et al.'s (2005) gamma statistic.

Figure 5 compares the five incongruence metrics examined in this study. There are strong correlations between symmetric and RF-distance, and between AU-test and SH-test results, but there is little correlation between these categories or with the gamma statistic. This provides a partial explanation for why the different tests identify different candidates for statistically significant incongruence.

**Figure 6:**
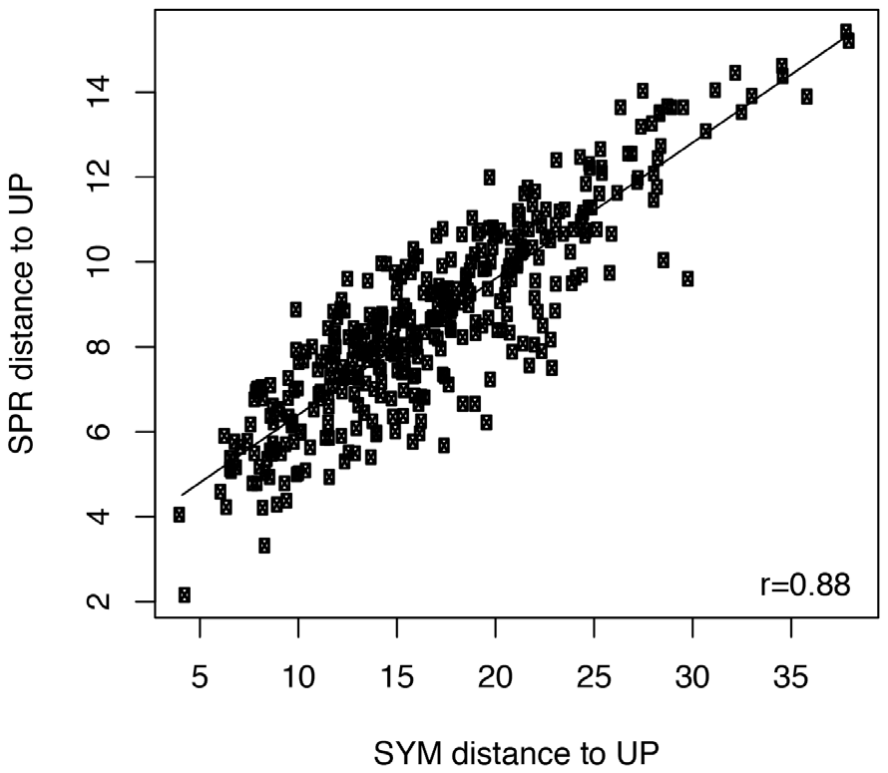
Correlation of symmetric distance to UP treecloud and SPR (MAST) distance to UP treecloud.

A deeper understanding of the behavior of Ge's gamma statistic in this dataset can be gained by examination of Figure 6. The figure shows the relationship of symmetric distance to UP treecloud and SPR (MAST) distance to UP treecloud. In this dataset, the two metrics are highly correlated. The strongest candidates for HGT, according to Ge's gamma, would have a high symmetric distance, combined with a low SPR distance; i.e., they would occupy the lower-right region of the plot, which is empty except for a few weak candidates at the edge.

### Statistical test of the centrality of the UP treecloud

**Figure 7:**
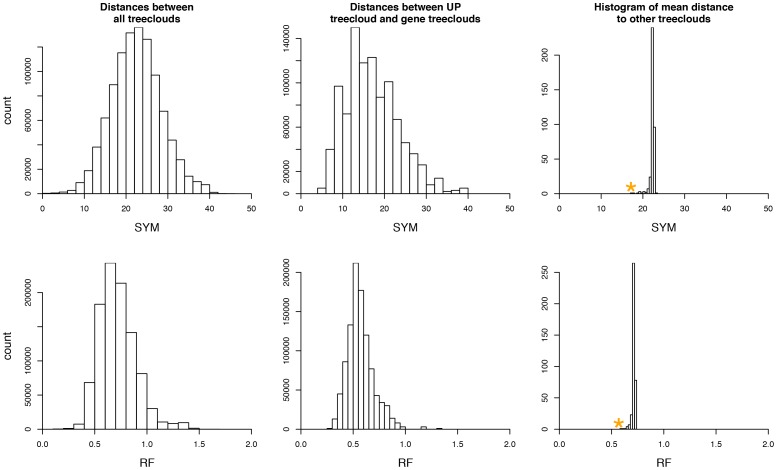
Distribution of distances between treeclouds- UP tree clouds included. Demonstration of the centrality of the UP treecloud in 380-dimensional treespace. Plots show histograms of the distances between treeclouds. Top: Symmetric distances (SYM). Bottom: RF-distances for rescaled trees. Left: Distribution of all distances between the treeclouds for all individual genes. Middle: Distribution of distances between the UP treecloud and all gene treeclouds. Right: 380 mean distances; for each treecloud, the average distance to all other treeclouds was calculated. Orange star: average distance from UP treecloud to all other treeclouds. For both types of distance metrics, the UP treecloud has the shortest mean distance to other treeclouds of all 380 treeclouds (p = 0.0026). The same result is achieved when all of the individual UP gene families are removed; the UP treecloud has the shortest mean distance to other treeclouds of 354 remaining treeclouds (p = 0.0028).

A central tendency of core gene treeclouds to cluster near the UP tree was observed by comparing the distances between all gene treeclouds and the distances between all gene treeclouds to the UP treecloud (Figure 7). For both the Symmetric and the Robinson-Foulds metric, there is a clear shift towards shorter distances for the UP treecloud-gene treecloud comparison. Furthermore, the average distance of each treecloud to one another showed that the UP tree statistically had the shortest mean distance (p = 0.0026) (Figure 7). This significant result was retained even when the UP treeclouds were removed from the calculation (Figure S7 in File S1; p = 0.0028).

The results of calculating the “treeishness” statistic, τ, are shown in Table 2. τ is approximate 0.65 regardless of the distance metric used, or the inclusion or exclusion of the individual UP protein families. The observed tau is statistically significantly different from 0 (no phylogenetic signal above random) and from 1 (meaning gene treeclouds identical to the species treecloud), although the p-values are many orders of magnitude lower for the former.

**Table 2:** Inputs and calculation of the “treeishness” statistic. p-values are the one-tailed probability of τ being equal or greater than one, or being equal or less than 0, respectively, given the observed mean and standard deviation of τ.

Treeclouds input	UPs?	mean gene-UP distance	mean gene-random distance	mean UP-random distance	treeishness (from means)	mean of treeishness array	SD of treeishness array	P(*τ*≥1)	P(*τ*≥0)
Symmetric	Yes	16.86	49.51	49.42	0.66	0.66	0.13	0.004	1.31E-07
RF	Yes	0.58	1.61	1.64	0.65	0.65	0.08	9.99E-06	3.54E-15
Symmetric	No	16.86	49.50	49.42	0.66	0.66	0.13	0.004	1.31E-07
RF	No	0.58	1.61	1.64	0.65	0.65	0.08	1.25E-05	7.33E-15

## Discussion

As Susko et al. [14] write, “it must be shown – not just assumed – that core genes do in fact share a common phylogenetic history.” Unlike many analyses, the present study provides positive support for the contention that the core gene dataset is significantly congruent. Furthermore, the results show that the core gene dataset has a central phylogenetic tendency that is best represented by the UP treecloud, whether or not the individual UP gene families are included in the analysis. The UP treecloud exists at or near the center of the treespace occupied by the gene treeclouds, and the gene treeclouds cluster around the UP treecloud much more closely than the distant tips-randomized treeclouds.

Methods of detecting HGT appear to often disagree, although this does not call into question the positive result of finding a central representative phylogenetic structure. Prosaic, non-HGT explanations of incongruence for certain metrics can be found, e.g. treeclouds derived from short alignments tend to have higher distances from the UP treecloud. None of the incongruence-detection metrics yielded a conclusion of strong evidence for widespread statistically significant incongruence, particularly not when Bonferroni corrections for multiple testing are applied. The statistic that is likely to be most sensitive to incongruence caused by HGT specifically, Ge's gamma, identifies only two gene families as potential HGT candidates.

Furthermore, what incongruence we detect seems to be fairly evenly distributed in terms of COGs and function; the candidates we pick up from all methods show no significant enrichment for any particular functional category. These findings may support the hypothesis that core genes are resistant to HGT, even in a group such as these marine cyanobacteria which has been thought to be subject to significant amounts of phage-induced HGT.

We therefore conclude that HGT is likely to be rare among the core genes of this cyanobacterial marine subclade, and that Ge et al.'s “cobweb of life” analogy – a backbone phylogenetic tree draped with relatively rare lateral transfer events, or at least rare lateral transfers between distance relatives – is likely to be more applicable to this dataset than models that suggest phylogenetic “tree” analogy is no longer useful or not likely to representative of more than a few percent of the genomic data. A more recent study [39] observes the lowest amount of HGT within the same marine subclade. Although they suggest that their low observations of HGT is due to their network analysis being based on simpler “presence/absence” data, our analysis is based on a more rigorous Bayesian phylogenetic analysis of all 379 core genes used in this study. Our results do not conflict with previous studies reporting large amounts of HGT reported within the cyanobacterial phylum [22], [39], [40], as our study is much more targeted to an ecologically distinct and monophyletic subclade of cyanobacteria. Although it is possible that large amounts of HGT may still exist within the phylum as a whole, the genes we consider here show little incongruence with one another, consistent with previous studies looking at the general trend of congruence between concatenated proteins, both for core and shell proteins from cyanobacteria [41].

Our results validate the suggestion of Hillis et al. [20] that tree-to-tree distances, displayed via NMMDS, would be a useful method for visualizing the relationships between tree clouds. The fact that there is a central phylogenetic tendency in the individual gene treeclouds, and that the UP treecloud plot at the center of this cluster, is immediately revealed by NMMDS plots, and confirmed by statistical tests. This central phylogenetic signal is most likely a reflection of the vertical history of genes, and thus supports previous efforts in finding vertical signal even in the face of pervasive HGT in bacteria [42]. It would appear from our data that the evolutionary history of Core Genes in marine cyanobacteria are relatively similar and cluster with the UP species tree, which can be interpreted as reflecting a substantial detectable history of vertical inheritance, thus supporting the Core Gene hypothesis in this group.

A novel feature of our study is the application of tree-distance methods to characterize the similarities and differences of Bayesian treeclouds, rather than individual trees. The availability of Bayesian posterior samples of trees enables analyses that are difficult or impossible with simpler analyses, because pairs of trees from within and between treeclouds can be sampled at will, which allows for the construction of null distributions and test statistics to assess whether or not observed distances are similar, whether or not treeclouds overlap or are truly distinct, etc.

This method is distinct from that advanced by Ane et al. [43], who used a Bayesian clustering method to identify a small set of topologies into which 106 treeclouds from 8 closely-related yeast species were probabilistically fit. This procedure then allowed estimation of a genome-wide concordance factor which estimated the proportion of the genome having each topology. This method is most applicable to situations where there is a strong prior expectation that there exists a small number of distinct true topologies, as is the case when incompletely lineages sorting is the major source of incongruence; this is unlikely to be the case in a system where rampant lateral transfer of individual genes is the hypothesis to be assessed.

One limitation of our methods is that they rely on having a dataset of core genes that exist in all taxa under study. Methods that take into account missing genes and gene duplications need more exploration. This is one major advantage of the neighbor-joining quartets method used in several papers [3], [14]. However, given our observation of the difficulty of avoiding incongruence due to prosaic causes such as alignment length and model misspecification (Text S1), even with a flexible and sophisticated Bayesian analysis method applied to a large group of taxa at once, quartets-based methods may be prone to these problems, since “some small trees (e.g., quartets) are among the hardest possible phylogenetic trees to estimate correctly” [44]. Doing a phylogenetic analysis of only 4 sequences at a time guarantees that difficult, long unbroken branches will often feature in the phylogenetic estimation problem; although admittedly researchers making use of this method take some steps to exclude ultra-long branches from their analysis. A better option might be to estimate phylogenetic gene trees in a traditional way, using all the available taxa at once, and then sample quartets from the resulting already-estimated tree or treecloud, and use these to quantify the signal in support of various vertical and horizontal inheritance hypotheses.

Based on our results, we encourage visualization of the treecloud-to-treecloud space as first, exploratory step for evaluating the “treeishness” of a multigene dataset. Such visualization steps are crucial to avoid being misled by rote application of statistical tests of simple and perhaps far-too-easily-refutable null hypotheses. As HGT is difficult to confidently detect except in evolutionarily recent cases and cases of HGT events crossing massive phylogenetic distances, NMMDS plots are a useful tool to allow the easy exploration of possible correlates of phylogenetic incongruence, such as alignment length and total treelength, which may otherwise be tempting to attribute to HGT. Finally, inclusion of “no phylogenetic signal” gene families in the visualization can give researchers a sense of the scale of incongruence in their gene trees. We are clearly in the age of thinking about phylogenetics as a process of statistical estimation rather than perfect reconstruction. Sources of incongruence are many, just as there are many sources of error for many measurements in many fields of biology, and even substantial amounts of incongruence might not be enough to totally obscure the signal of vertical ancestry. Therefore methods should be used that will detect the signal of vertical treelike inheritance, even if it exists amongst substantial noise from various sources.

A promising approach towards taking into account the major sources of incongruence between organismal trees and gene trees is suggested by Bousseau et al. [45], who incorporate parameters for gene duplication, gene deletion, HGT, and incomplete lineage sorting, along with the standard sequence evolution parameters such as substitution model and base frequencies. However, the computational effort required for this sort of approach is extreme, which may limit its applicability for problems such as commonly experienced in genomics, where hundreds of homologous genes are available for species in a closely-related clade, and a first assessment of the possibility of obtaining an organismal tree with reasonable accuracy is desired.

Our results give evidence that, in the case of the cyanobacterial groups under consideration, it should be possible to estimate organismal trees. Furthermore, estimating organismal trees should be possible in many situations where individual gene trees have an evident central phylogenetic tendency, whether or not these genes are part of the “tree of one percent” [9]. It seems likely that the “tree of one percent” may represent much of the history of a substantially larger percentage of genes, as well as the organismal tree.

## Supporting Information

File S1 **Supplemental Information on: Complexity Hypothesis background, methods, and results; regression analysis; and supplementary figures. Included in File S1: Figure S1. Example gene trees for single-gene alignments. Figure S2. Example NMMDS plot showing the location of randomly-selected treeclouds subjected 1, 3, 6, or 28 (complete randomization) switches of labels. Figure S3. Functional Categories of HGT Candidates. Figure S4. NMMDS plots with treeclouds colored by value. Figure S5. Correlation of treelength with alignment length, unscaled RF distance to UP tree, symmetric distance to UP tree, and rescaled RF distance to UP tree. Figure S6. Negative correlation of alignment length and distance to the UP treecloud. Figure S7. Distribution of distances between treeclouds- UP tree clouds excluded. Text S1. Complexity Hypothesis background; Complexity Hypothesis: Methods and Results; Functional Categories of HGT candidates; Effect of alignment length and divergence on tree distance: Methods and Results; and Regression Analyses.**
(DOC)
