# Supplementary material for: Bayesian Analysis of Congruence of Core Genes in Prochlorococcus and Synechococcus and Implications on Horizontal Gene Transfer
Source: PLoS One. 2014 Jan 21;9(1):e85103. doi: 10.1371/journal.pone.0085103 (PMC3897415; doi:10.1371/journal.pone.0085103)
Supplement: File S1 — Supplemental Information on: Complexity Hypothesis background, methods, and results; regression analysis; and supplementary figures. Included in File S1: Figure S1. Example gene trees for single-gene alignments. Figure S2. Example NMMDS plot showing the location of randomly-selected treeclouds subjected 1, 3, 6, or 28 (complete randomization) switches of labels. Figure S3. Functional Categories of HGT Candidates. Figure S4. NMMDS plots with treeclouds colored by value. Figure S5. Correlation of treelength with alignment length, unscaled RF distance to UP tree, symmetric distance to UP tree, and rescaled RF distance to UP tree. Figure S6. Negative correlation of alignment length and distance to the UP treecloud. Figure S7. Distribution of distances between treeclouds- UP tree clouds excluded. Text S1. Complexity Hypothesis background; Complexity Hypothesis: Methods and Results; Functional Categories of HGT candidates; Effect of alignment length and divergence on tree distance: Methods and Results; and Regression Analyses. (DOC) [file pone.0085103.s001.doc]

**File S1. Supplemental Information** on: Complexity Hypothesis background, methods, and results; regression analysis; and supplementary figures.

**Bayesian Analysis of Congruence of Core Genes in *Prochlorococcus* and *Synechococcus* and Implications on Horizontal Gene Transfer**

**Nicholas J. Matzke^1^†‡, Patrick M. Shih^1^¶, Cheryl A. Kerfeld*¶§**

**^1^**Authors contributed equally to this work. †Department of Integrative Biology, University of California, Berkeley, United States; ‡Current position: National Institute for Mathematical and Biological Synthesis, University of Tennessee, Knoxville, United States; ¶Department of Plant and Microbial Biology, University of California, Berkeley; §US Department of Energy-Joint Genome Institute; *Corresponding author, 2800 Mitchell Drive, Walnut Creek, CA 94597; ckerfeld@lbl.gov.

**Supplemental Text**

**Complexity hypothesis: Introduction**

The Core Gene Hypothesis does not itself provide a reason for the decreased frequency of HGT amongst Core Genes. However, the Complexity Hypothesis, as put forth by Jain et al., suggests that proteins involved in many conserved protein-protein or protein-macromolecule interactions are less likely to be transferred . This hypothesis predicts that it will not usually be selectively advantageous for a foreign gene to take the place of an endogenous one, as the protein has not coevolved with the other components that the native protein interacts with. Thus, the more complex the interactions, the less likely the event of HGT. Specifically, genes encoding for informational processes, such as proteins involved in cellular processes like transcription and translation, have been hypothesized to have a decreased chance of HGT because they are often found in large complexes involving many other protein subunits and macromolecules (ie. ribosomal machinery) .

**Complexity Hypothesis: Methods and Results**

**Functional Categories of HGT candidates.** Based on the complexity hypothesis, it is thought that informational proteins, or proteins involved in complex multi-subunit machineries will be less likely to undergo HGT. Core gene trees with large between-treecloud distances exhibit the most phylogenetic incongruence with the UP tree, and thus might be considered the best candidates for potential HGT events. Under the Complexity Hypothesis, it might be expected that the genes with maximal incongruence with the UP tree would be genes less involved in interactions with multiple other proteins. We took the top 5^th^ percentile of core genes based on between-treecloud distances and characterized each gene based on its COG family. However, based on candidates from likelihood- and distance-based methods, there is no observed strong bias for any functional group (including Informational Storage Proteins) (Supplementary Figure S3, and summarized in Supplementary Table S1), and thus our data does not lend support to the complexity hypothesis.

**Effect of alignment length and divergence on tree distance: Methods and Results**

To investigate possible non-HGT sources of incongruence, we examined three variables that might explain variability in treecloud structure: (1) Average treelength of (raw, unscaled) trees in the treecloud. The treelength is the sum of the lengths of all of the branches in a tree, and branch lengths represent expected amount of substitution per site on the branch, so total treelength should correlate tightly with measures such as average percent difference in sequences in a protein family. (2) Alignment length, which is a direct measure of the amount of sequence information available for the MrBayes analysis of each gene family. Finally, (3) alignment length divided by average treelength. Since longer alignments have provide more sequence information with which to estimate the model parameters and the tree, and shorter trees likely have less homoplasy and retain more phylogenetic signal than longer trees, it might be the case that this ratio correlates with congruence more than the others.

A clear picture is presented in the upper left plot of Supplementary Figure S4, where the treecloud is colored by average total treelength of the trees in the each treecloud. As expected, the short trees cluster with the overall slowly-evolving sequence of the UP tree. When the effect of raw treelength is removed by looking at NMMDS plots of symmetric distance or RF branchlength distance on rescaled trees, the strong clustering of short trees at the center of the cluster is not observed. If anything, treeclouds with short trees are found at the periphery of the symmetric distances NMMDS plot. It might be the case that some very slowly-evolving proteins have experienced so few substitutions in this clade that some short branches are not well-recorded. The NMMDS plot of RF distance on rescaled treeclouds (Supplementary Figure S4, upper right), interestingly, may give some evidence for this hypothesis. Despite the fact that all of the trees in the plot are of length 1, the coloring of the treeclouds with the corresponding raw, unscaled tree distances seems to show that shorter treelengths plot in the lower right and longer treelengths plot in the upper left. In other words, short trees seem to have relative proportions similar to other short trees, and long trees seem to have relative proportions similar to other long trees. Intermediate treelengths are found roughly in the center of the cluster with the UP tree, supporting the idea that treeclouds with intermediate treelengths may best estimate the true underlying tree. Admittedly, however, these are noisy patterns with many exceptions, and need to be investigated further.

Turning to alignment length (Supplementary Figure S4, second row), all of the plots show that shorter alignments (red) tend to be spread around the periphery of the treespace, with long alignments (yellow) near the center of the cluster and the UP treecloud. This is readily understandable as the result of shorter alignments having less information, and thus being less accurate at estimating the true tree. However, it is also worth noting that comparison of the leftmost plots in the first and second row of Supplementary Figure S4 shows that there seems to be an unanticipated correlation between alignment length and treelength. I.e., short trees seem to be produced by small alignments (although there are many exceptions). Another way of saying it is that amongst the 379 gene families under analysis, there are a substantial number of proteins that are both small and relatively slowly evolving.

Perhaps because of this unanticipated correlation, coloring the NMMDS plots by alignment length / treelength (here, yellow would indicate a large, slowly-evolving alignment, which is presumably the most informative) does not show much of a pattern (except for RF distances on unscaled trees, which still shows the influence of the treelength effect).

**Regression analyses.** Use of scatterplots and regression analysis allows us to test the inferences made based on observations of the NMMDS plots. Supplementary Figure S5 shows the correlation of the alignment length and the tree distance metrics with treelength. Correlations are weak except for that for RF-distance on unscaled trees, where differences in treelength dominate. Contrary to expectations, treeclouds with shorter treelengths tend to have somewhat more incongruence with the UP tree than average (Supplementary Figure S5, bottom row). On the other hand, Supplementary Figure S6 confirms the conclusion that longer alignments tend to produce treeclouds with more congruence with the UP treecloud.


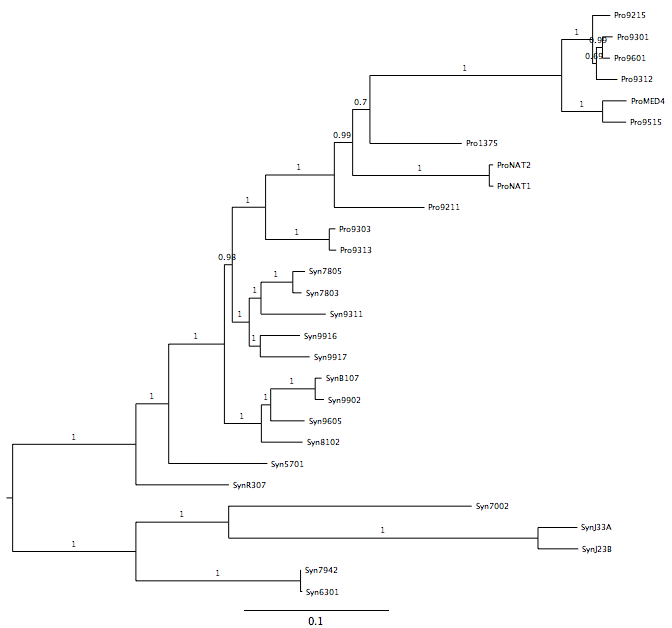


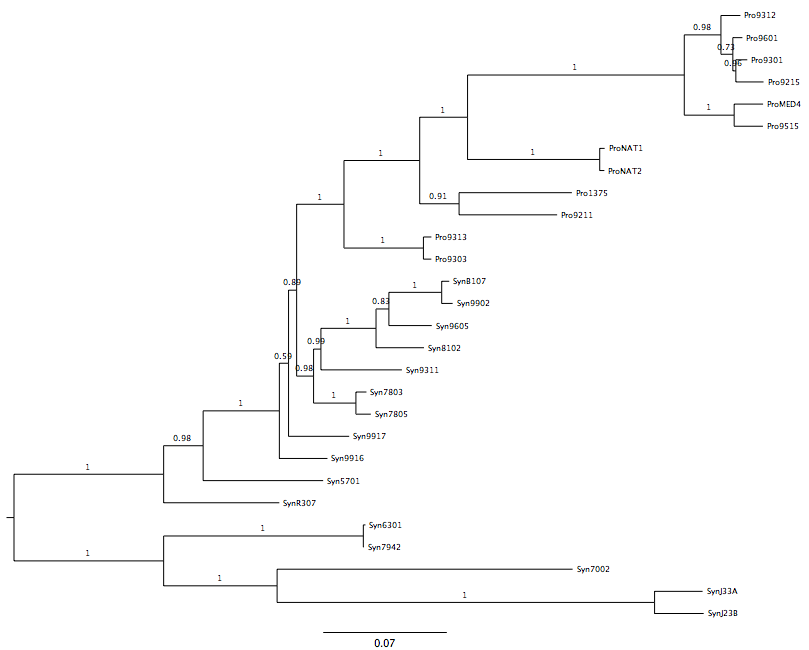


**Supplementary Figure S1. Example gene trees for single-gene alignments.** Shown here: gene tree #1 (DNA polymerase III subunit beta) and gene tree #10 (elongation factor P).


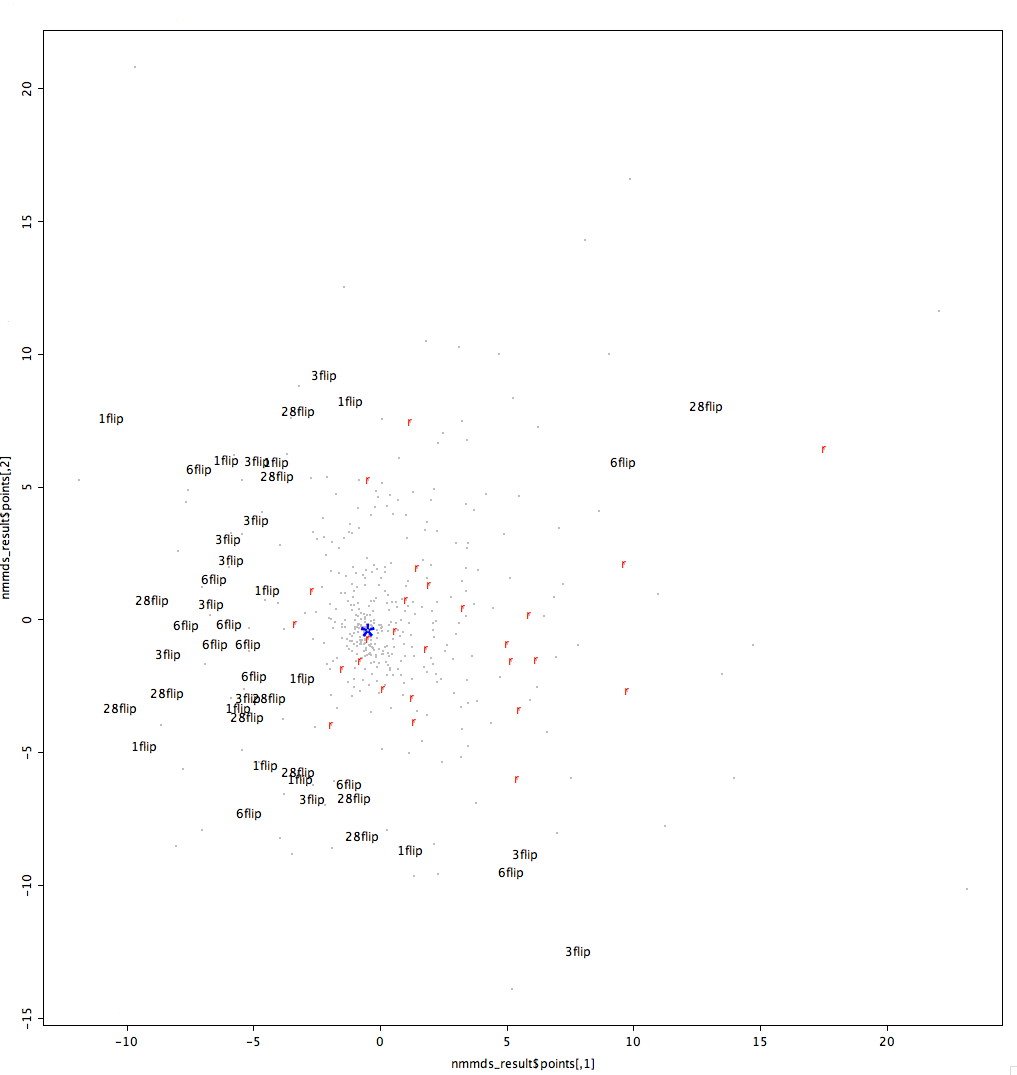


**Supplementary Figure S2.** Example NMMDS plot showing the location of randomly-selected treeclouds subjected 1,3, 6, or 28 (complete randomization) switches of labels.


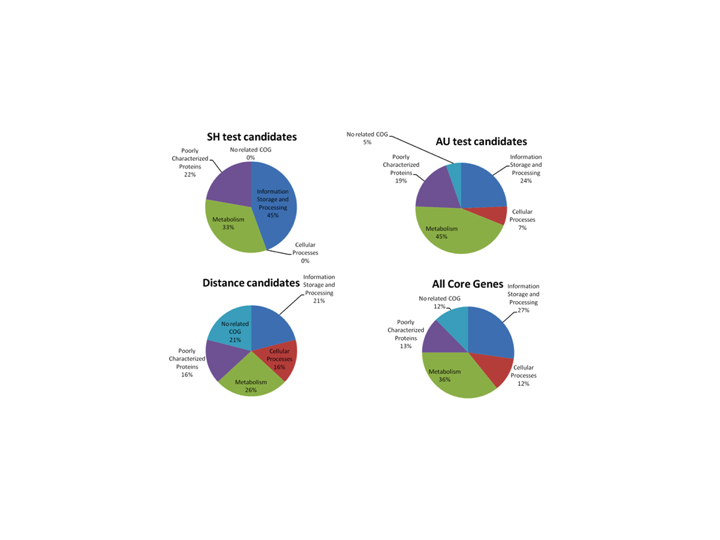
**Supplementary Figure S3. Functional Categories of HGT Candidates.**

**
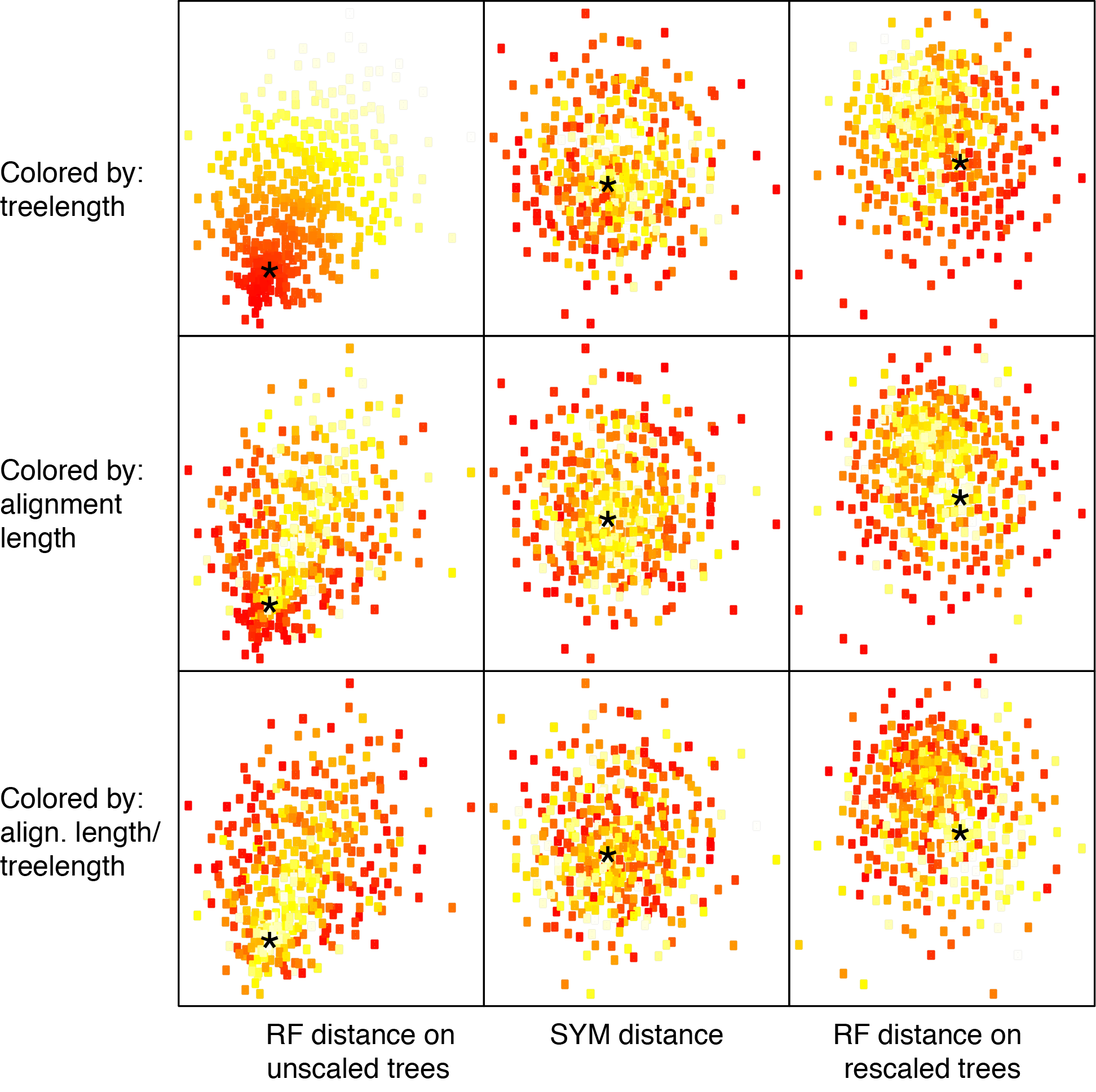
**

**Supplementary Figure S4. NMMDS plots with treeclouds colored by value.** Plots derived from the different treecloud-to-treecloud distances (columns), colored by treecloud average treelength (row 1), alignment length (row 2), and alignment length divided by treelength (row 3). Color scheme: Red=low values, yellow=high values. Black star represents the UP tree. The NMMDS plots derived from RF branchlength distances on unscaled trees are dominated by the treelength effect. Both symmetric distance and RF distance on rescaled trees show a pattern wherein long alignments tend to cluster in the middle of the treespace, near the UP treecloud, whereas short alignments tend to occur at the periphery.


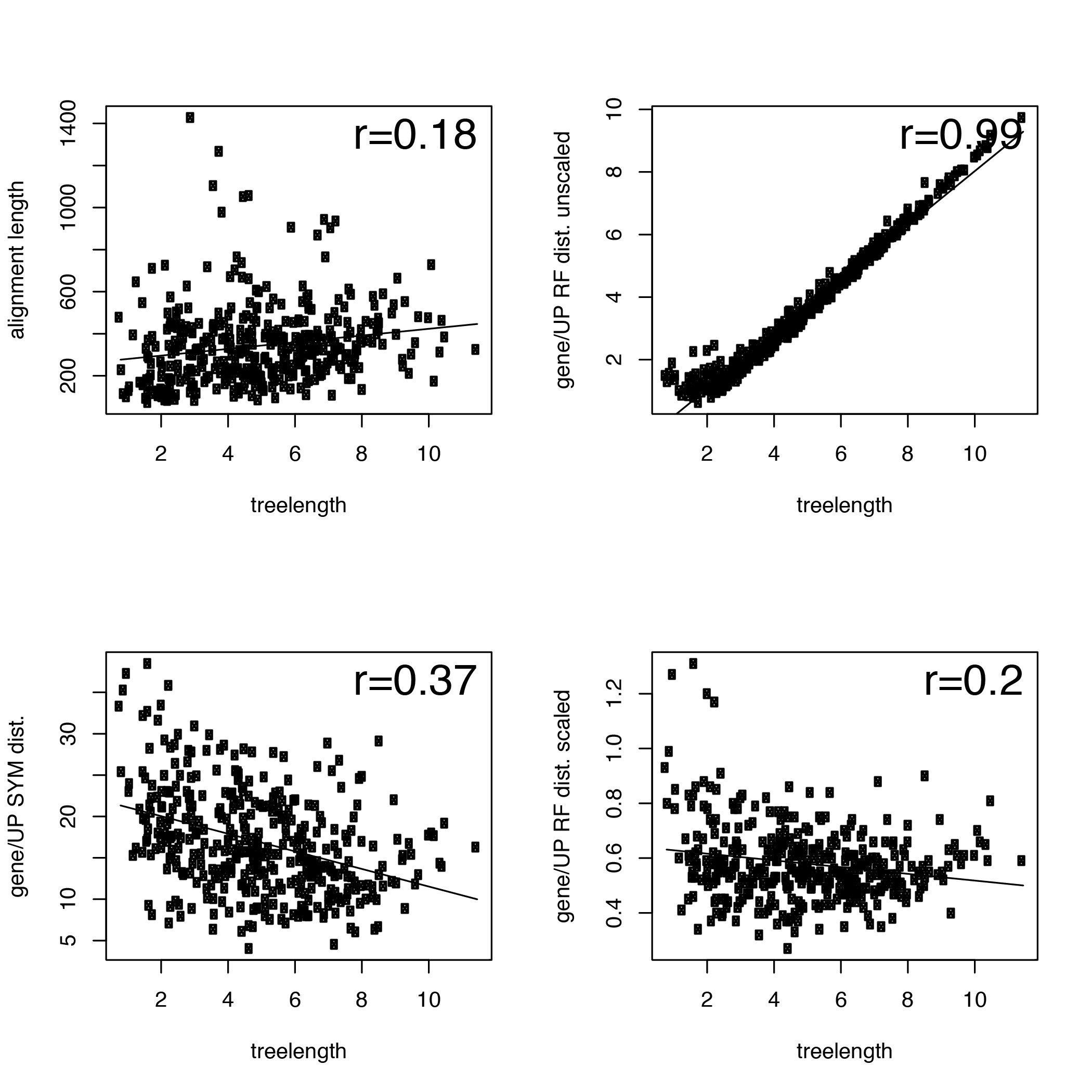


**Supplementary Figure S5.** Correlation of treelength with alignment length, unscaled RF distance to UP tree, symmetric distance to UP tree, and rescaled RF distance to UP tree. Treelength dominates the unscaled RF distance metric.


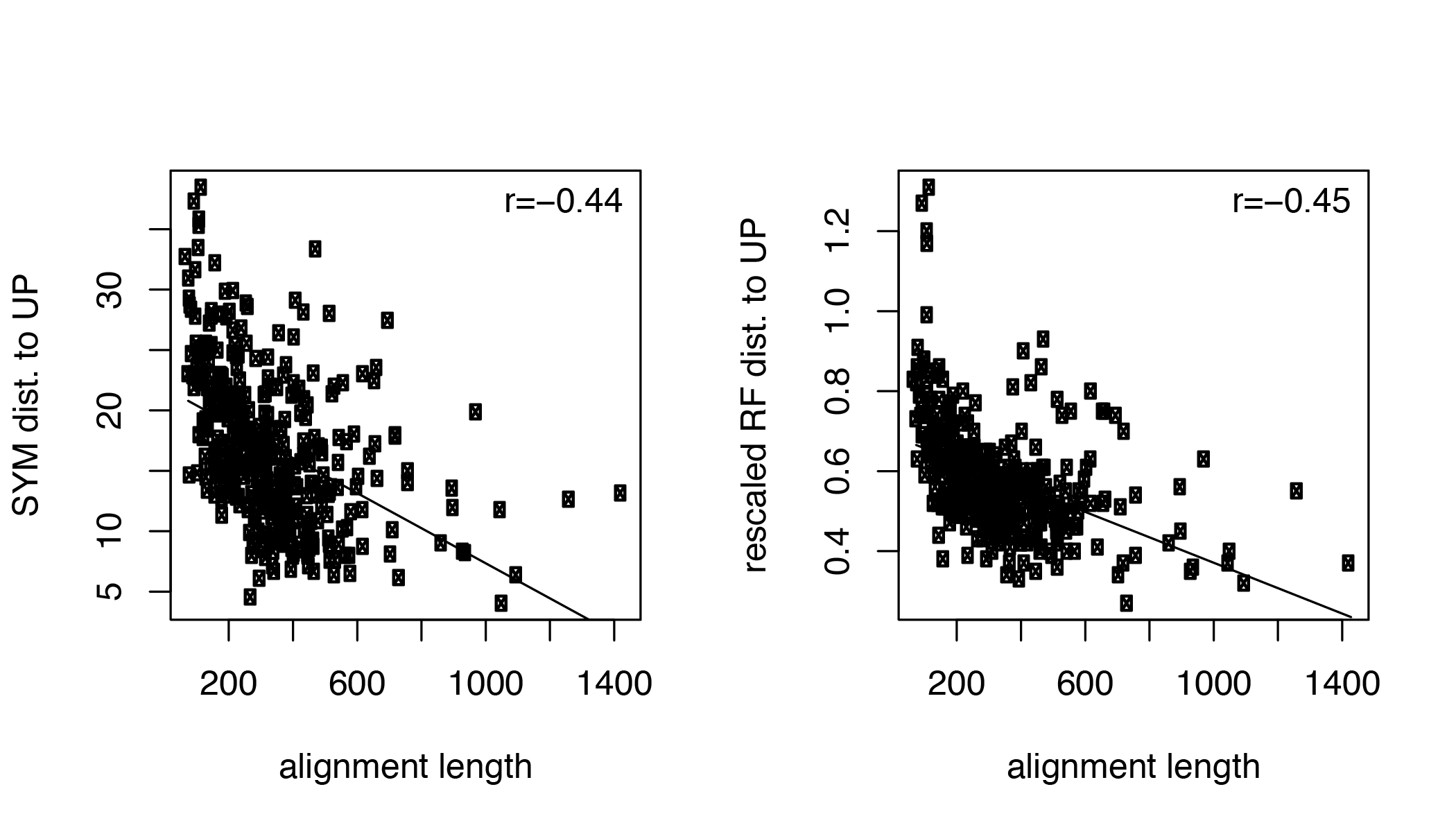


**Supplementary Figure S6. Negative correlation of alignment length and distance to the UP treecloud.**


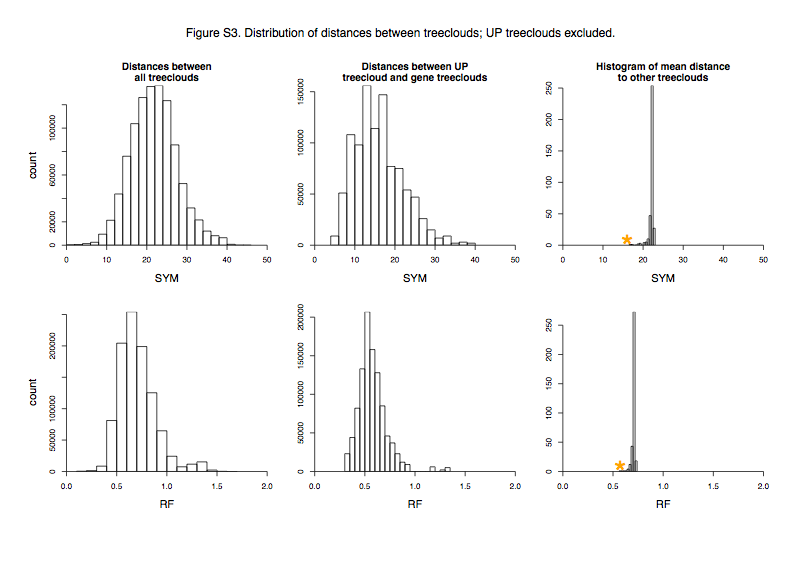


Supplementary Figure S7. Distribution of distances between treeclouds- UP tree clouds excluded. The analysis of Figure 4 repeated after the removal of the individual UP treeclouds. The pattern remains that the concatenated UP treeclound is on average closer to the individual gene treeclouds than the average distance of individual gene treeclouds to each other. The concatenated UP treecloud has the shortest mean distance to other treeclouds of 354 remaining treeclouds (p=0.0028).

**Supplemental References**

1. Jain R, Rivera MC, Lake JA (1999) Horizontal gene transfer among genomes: The complexity hypothesis. Proc Natl Acad Sci USA 96: 3801-3806.

2. Rivera MC, Jain R, Moore JE, Lake JA (1998) Genomic evidence for two functionally distinct gene classes. Proc Natl Acad Sci USA 95: 6239-6244.
